# Change in gliding properties of the iliotibial tract in hypermobile Ehlers–Danlos Syndrome

**DOI:** 10.1007/s40477-023-00775-7

**Published:** 2023-02-19

**Authors:** Tina J. Wang, Antonio Stecco, Robert Schleip, Carla Stecco, Carmelo Pirri

**Affiliations:** 1 Department of Physical Medicine & Rehabilitation, Loma Linda University School of Medicine, Loma Linda, CA USA; 2 Department of Rehabilitation Medicine, New York University School of Medicine, New York, NY USA; 3 Department of Conservative and Rehabilitive Orthopaedics, Technical University of Munich, Munich, Germany; 4 Department of Neurosciences, Institute of Human Anatomy, University of Padova, Padua, Italy; 5 429 N Central Ave, Upland, CA 91786 USA

**Keywords:** Ehlers–Danlos syndrome, Shear strain, Ultrasonography, Fascia, Iliotibial tract

## Abstract

**Purpose:**

Fascial changes in hypermobile Ehlers–Danlos syndrome (hEDS), a heritable connective tissue disorder, can be used visualized with sonoelastography. The purpose of this study was to explore the inter-fascial gliding characteristics in hEDS.

**Methods:**

In 9 subjects, the right iliotibial tract was examined with ultrasonography. Tissue displacements of the iliotibial tract were estimated from ultrasound data using cross-correlation techniques.

**Results:**

In hEDS subjects, shear strain was 46.2%, lower than those with lower limb pain without hEDS (89.5%) and in control subjects without hEDS and without pain (121.1%).

**Conclusion:**

Extracellular matrix changes in hEDS may manifest as reduced inter-fascial plane gliding.

## Introduction

In musculoskeletal radiology, ultrasonography is a powerful tool for the diagnosis of many pathologic conditions affecting the myofascial system including the fascia. More superficial fascial structures like the iliotibial tract (ITT) can be easily studied with ultrasonography [1].

Given its cost-effective real-time noninvasive nature, ultrasonography is the first-choice technique in the diagnosis of fascial based pathology [1]. Hypermobile Ehlers–Danlos syndrome (hEDS) is the most common type of EDS and is a heritable connective tissue disorder characterized by changes in the extracellular matrix (ECM) [2, 3] of the fascial system that can be visualized under ultrasonography [4]. These changes in the ECM may correlate with the high level of myofascial pain in hEDS [4, 5].

Prior ultrasound studies of the iliac fascia and ITT did not demonstrate a difference in thickness in those with hEDS compared with groups without hEDS. Non-hEDS subjects with pain had a higher strain index (more softening of the fascia with relative stiffening of the muscle) compared with hEDS subjects and non-hEDS subjects without back or knee pain. In myofascial pain, softening of the fascia may occur from increase in ECM content and relative increase in stiffness of the muscle; this change is not as pronounced in hEDS [4].

Prior studies have looked at inter-fascial gliding characteristics in normal populations and in populations with back pain [6–8]. To date, there are no published data regarding inter-fascial plane gliding in hEDS. Therefore, the objective of this study was to characterize inter-fascial plane gliding in hEDS patients and introduce the use of dynamic ultrasonography to assess dynamic fascial movement in this population.

## Methods

This study was performed in accordance with ethical standards on human experimentation and with the Helsinki Declaration of 1975, as revised in 1983 and approved by Institutional Review Board (Study 2019-92-CAS). The investigation and use of patient data for research purposes were in accordance with the Declaration of the World Medical Association. Written informed consent was obtained for the 9 adult patients (≥ 18 years of age) who were examined prospectively.

The 3 subjects with knee pain without hEDS had diagnoses of medial compartment osteoarthritis with associated patellar tendonopathy, medial collateral ligament sprain, and patellofemoral ligament sprain as diagnosed by physical examination and MRI. Three control subjects did not have hEDS or knee pain. The 3 subjects with hEDS had diffuse pain including lower limb pain as stipulated by the 2017 hEDS diagnostic criteria [9]. All subjects consented to ultrasound examination at an outpatient Physical Medicine & Rehabilitation practice.

**Fig. 1:**
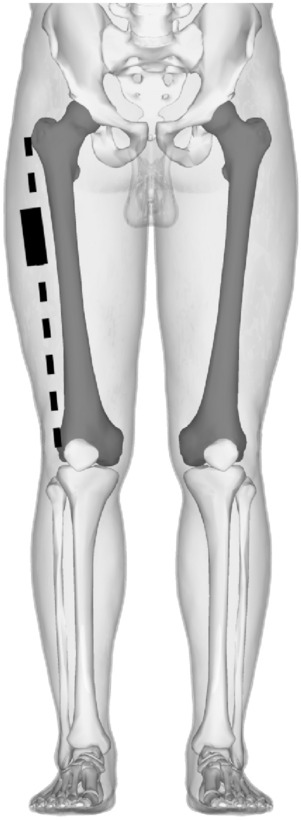
The ultrasound transducer (represented by the black rectangle) was placed longitudinally at the proximal one-third distance between the femoral condyle and the greater trochanter of the right lower limb

The ITT of the right lower limb was examined with B-mode scanning using the Sonimage^®^ HS-1 (Konica Minolta Corporation, Japan) and a L18-4 transducer. The patients were examined in a relaxed supine position with arms relaxed to the sides on the examination table. The ultrasound transducer was placed longitudinally at the proximal one-third distance between the femoral condyle and the greater trochanter of the right lower limb (Fig. 1). The transducer was moved until the direction of the perimysium was parallel to the transducer and over the area of interest—at the merger of the superficial, intermediate, and deep layers of the ITT [10]. The transducer was lightly stabilized by hand taking great care not to compress the tissues at any time. Approximately 100 ml of gel used between transductor and skin.

**Fig. 2:**
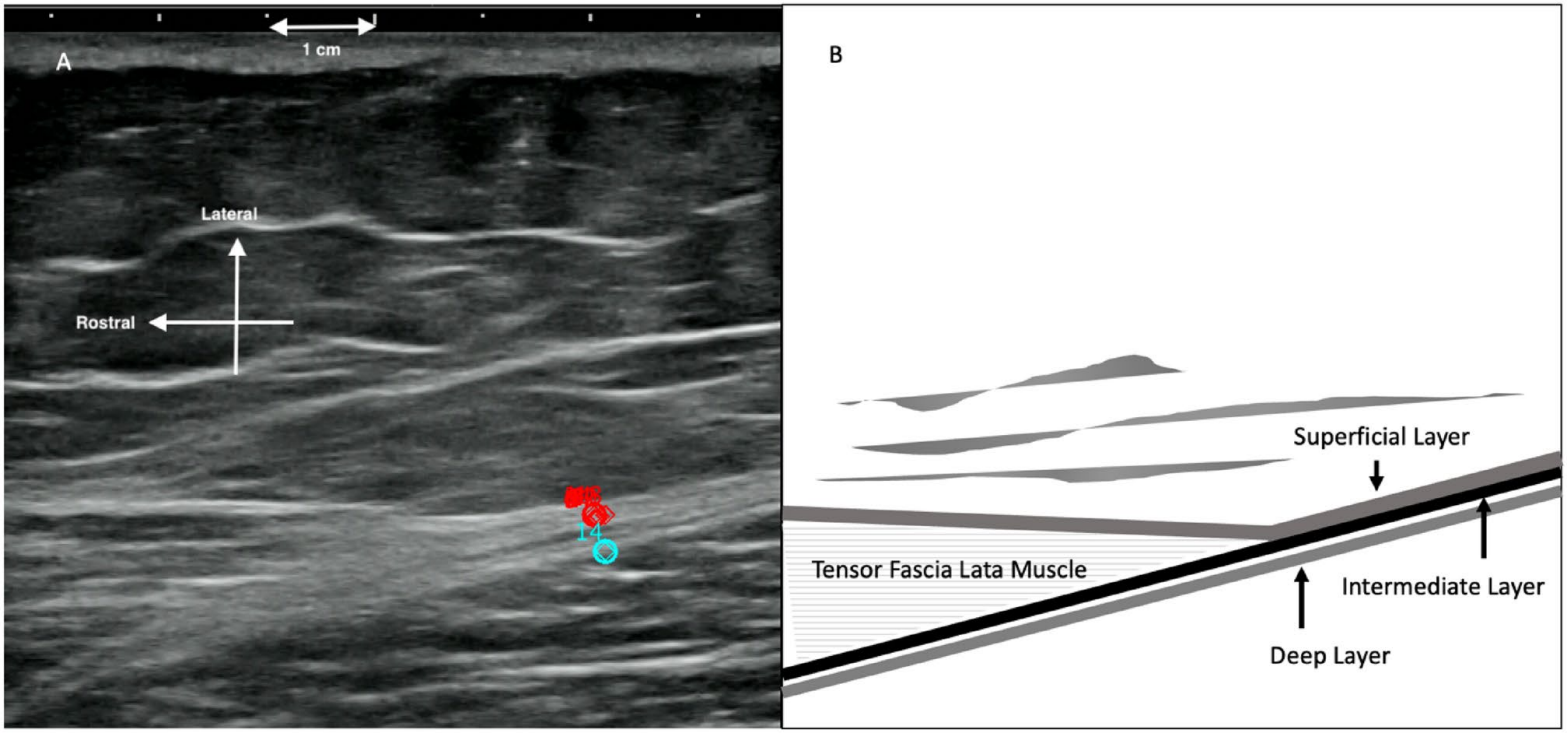
**A** B-mode (and still frame of cine loop) ultrasound image from the right lower limb. Rostral-caudal displacement (tissue motion) between two successively acquired ultrasound frames captured at 40 ms time lapse was computed for each successive pair of ultrasound frames in a 1 × 1.5 cm region of interest (ROI) centered on a sub-ROI. The superficial sub-ROI was centered on the merged fascia of the superficial and intermediate layers of the iliotibial tract (top circle in figure). The deep sub-ROI was centered on the deep layer of the iliotibial tract (bottom circle in figure). **B** Schematic of anatomical structures

Ultrasound cine-recording of the motion was acquired of the ITT during plantar flexion and dorsiflexion of the ankles to assess for fascial displacement and myofascial displacement from a distal site [11]. The ITT is continuous with the crural fascia, a thick lamina of connective tissue that envelopes the lower leg from the knee to the retinaculum of the ankle [12]. Ultrasound data from right lower limb were processed with Tracker (Natick, MA), a modeling tool built on the Open Source Physics Java framework. Tissue displacements between successive ultrasound frames were estimated from raw ultrasound data using cross-correlation techniques [6] using the method detailed by Dones et al. [13]. Rostral-caudal displacement (tissue motion) between two successively acquired ultrasound frames captured at 40 ms time lapse was computed for each successive pair of ultrasound frames in a 1 × 1.5 cm region of interest (ROI) centered on a sub-ROI. The superficial sub-ROI was centered on the merged fascia of the superficial and intermediate layers of the ITT. The deep sub-ROI was centered on the deep layer of the ITT (Fig. 2).

The cumulative lateral strain between superficial and deep sub-ROIs was calculated through one plantarflexion/dorsiflexion cycle. Shear strain between the sub-ROIs was calculated as the absolute difference in caudal-cephalad direction motion between the superficial and deep sub-ROIs divided by the distance (2 mm) [1] between the centers of the two sub-ROIs and expressed as a percentage [6].

The maximum change in length was calculated by the maximum change in relative tissue movement in a rostral-caudal direction in one plantarflexion/dorsiflexion cycle [14]. Specifically, this was the maximum length change between the superficial and deep sub-ROIs within one plantarflexion/dorsiflexion cycle. The sub-ROIs are shown in Fig. 2A.

## Results

The average age was 42.1 years. Seven subjects were females. The demographics and results are reported in Table 1. The average maximum change in relative tissue displacement between the deep and superficial layer of the ITT was 1.3 mm in the hEDS group, 2.9 mm in the knee pain without hEDS group, and 4.8 mm in the control group. The average shear strain was 46.2% in hEDS subjection, 89.5% in subjects with lower limb pain without hEDS, and 121.1% in subjects without hEDS or lower limb pain. In the hEDS subjects, the shear strain was 50% lower than those with lower limb pain without hEDS and 25% lower in control subjects without pain or hEDS.

**Table 1:** Demographic and results of ultrasound measurements

hEDS	Age (years)	Body mass index	Shear strain (median)	Maximum change in length in mm (median)
hEDS average	31.3 (30)	19.7 (18.6)	46.2% (28.2%)	1.3 (1.1)
Non-hEDS with knee pain average	63.7 (69)	23.7 (24.5)	89.5% (92.8%)	2.9 (3.0)
Control average	31.3 (32)	19.4 (19.0)	121.10% (110.0%)	4.8 (5.7)

*hEDS* hypermobile Ehlers–Danlos Syndrome, *mm* millimeters

## Discussion

The purpose of this study was to characterize inter-fascial movement in hEDS population using musculoskeletal ultrasonography. Currently, hEDS is diagnosed clinically and adjunctive ultrasound characterization may be helpful in clinically unclear cases.

Previous ultrasound studies showed that although the ITT did not demonstrate a statistically significant difference in thickness in those with hEDS compared with groups without hEDS, there was an observable overall increase in thickness of the ITT in hEDS subjects that approached statistical significance. There was also a higher strain index (more softening of the fascia with relative stiffening of the muscle) compared with non-hEDS subjects without knee pain [4]. This suggests that softening of the fascia may occur from increase in ECM content that may affect gliding and lead to myofascial pain [5].

Ultrasound examination of inter-fascial movement at the ITT in hEDS showed that hEDS had significantly less movement compared to their counterparts without hEDS. Prior studies of healthy adult males suggested that reduced inter-fascial plane movement was associated with reduced joint flexibility [15]. In contrast, the results of this study suggest in pathologic states of increased joint flexibility in hEDS, fascial displacement may be reduced.

Changes in inter-fascial plane gliding characteristics may occur in hEDS from pathologic changes in matrix metalloproteinases in the ECM [16] and lead to subsequent alteration of other ECM components, resulting in increased viscosity, and reduced lubrication and sliding movement of fascia [7]. Alteration in αvβ3 integrin-ILK complexes in focal adhesions may also occur from the fibroblast-to-myofibroblast transition [17]. Alterations of gliding interactions may influence joint mechanics and lead to impaired biomechanics, proprioceptive dysfunction, pain [7, 8] and predisposition to injuries [18, 19]. The low shear strain in hEDS compared with non-hEDS counterparts suggest that this process may be amplified in hEDS and may be associated with the high prevalence of joint instability [20] and proprioceptive dysfunction [21, 22].

Prior studies have demonstrated reduced stiffness of tendon, fascia and muscle in hEDS/hypermobile spectrum disorders [4, 23, 24]. This study shows that inter-fascial gliding may also be impaired and may correlate with the high prevalence of knee pathology (17.7–26%) [20] and pain seen in the hEDS population. Prior animal study has shown that connective tissue contained in muscle may account for 30% of muscle force production [25]**.** Overall, changes in the fascia stiffness and gliding in hEDS may correspond to the 30–49% reduction in strength seen in this population [22, 26].

The limitation of this study was the small sample size. Ultrasound is a semi-quantitative methodology and dependent on operator experience. Baseline characteristics of ITT movement between layers may be variable within normal populations and larger studies are needed [27]. Nevertheless, despite the preliminary nature of our data, this study highlights the importance of ultrasonography in the examination of fascia characteristics in the hEDS population. Although the number of cases is small, the data presented are novel and can serve as baseline data to guide future larger scale studies. Further prospective research with a larger number of participants across practice settings is recommended.

## Conclusion

This analysis provides insight into fascial characteristics in hEDS using ultrasonography. hEDS patients exhibit decreased inter-fascial shear strain and total movement between the deep and superficial layers of the ITT compared to non-hEDS counterparts. In non-hEDS subjects with lower limb pain, a decrease in inter-fascial shear strain is present and is more pronounced in hEDS subjects. Increase in ECM content may be associated with alteration in gliding properties leading to pain, joint instability, and dysfunction.

## Data Availability

Data is avaible from the authors by request.
